# Delayed Treatment of Traumatic Cervical Dislocation: A Case Report and Literature Review

**DOI:** 10.1155/2022/7756484

**Published:** 2022-03-02

**Authors:** Fabian Roland Bechet, Pierre Stassen, Dan Scorpie, Thierry Della Siega

**Affiliations:** From the Orthopaedic and Trauma Surgery Department, Les Cliniques du Sud-Luxembourg-Vivalia, Arlon, Belgium

## Abstract

Neglected unreduced cervical dislocation is very uncommon. In our case (a lady who stayed asymptomatic for 13 months before development of cervicobrachialgia), the anterior reduction/arthrodesis was easy, and we did not find any benefit from an additional posterior procedure thanks to a congenital block between C7 and T1 vertebral bodies. This point is nevertheless a matter of debate. After a review of the literature, we did not find any consensus about the ideal scheme and sequence to reduce and stabilize this delayed type of cervical trauma. We emphasize the need of dynamic radiographies to exclude unstable injuries but also a prereduction MRI (especially in unexaminable patients) to detect any dangerous disc fragment. If there is no visible change in the radiological status while attempting to reduce the dislocation by external maneuvers, there is little chance to reduce it successfully only by a single approach. Therefore, in irreducible delayed dislocations, it seems safer to prepare the reduction/fusion stage (either anterior/posterior, depending on the habits and skills of the surgeon) by a first stage carrying out a release of the fibrous tissues on the opposite side (either posterior to release the facet joints or anterior to release the intervertebral disc), followed by the reduction/fusion stage itself and then by a third stage to lock the level. Like many authors, we recommend an anterior approach first in case of an extruded disc visible on the MRI, and therefore, we show a preference for the anterior-posterior-anterior sequence in irreducible delayed cervical dislocations.

## 1. Introduction

There is little in the literature about neglected asymptomatic unreduced cervical dislocation. The delay for the diagnosis is probably due to incomplete plain X-rays because of technical difficulties in visualizing the entire cervical spine in the trauma patients. We report here a very uncommon case of complete dislocation of the C6-C7 level, completely free of neurological symptoms, for more than one year after a fall.

## 2. Case Report

**Figure 1:**
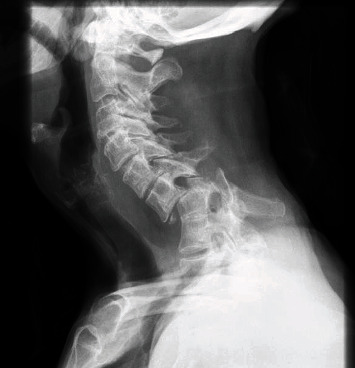
Preoperative X-rays (lateral view).

A 70-year-old lady was referred for bilateral radicular pain in the upper limbs since one week. The initial examination did not show any motor or sensitive deficit. Plain X-rays of the cervical spine (Figure 1) showed a delayed unreduced anterior dislocation at the C6-C7 level. She actually fell backwards 13 months ago with her occiput having hit a table. Nevertheless, she did not consult any clinician afterwards as she had no complaints but mild cervical pain.

A CT scan confirmed the longstanding pattern of the dislocation with chronic changes in the cortical layer of the two vertebras.

**Figure 2:**
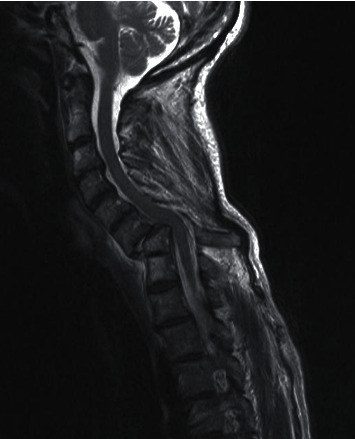
Preoperative MRI scan (sagittal cut).

An electromyogram did not show any deficit but some chronic abnormalities in the C7 nerve roots. An MRI scan (Figure 2) did not show any compression of the spinal cord and no prolapsed disc but a bayonet-shaped spinal canal because of the C6 anterior slip. As no motor deficit was observed, she was enlisted for a reduction with stabilization the next week.

Four days later, she unfortunately developed a tetraparesia with inability to stand up due to an acute weakness in the four limbs. The operation was then carried out as an emergency case.

**Figure 3:**
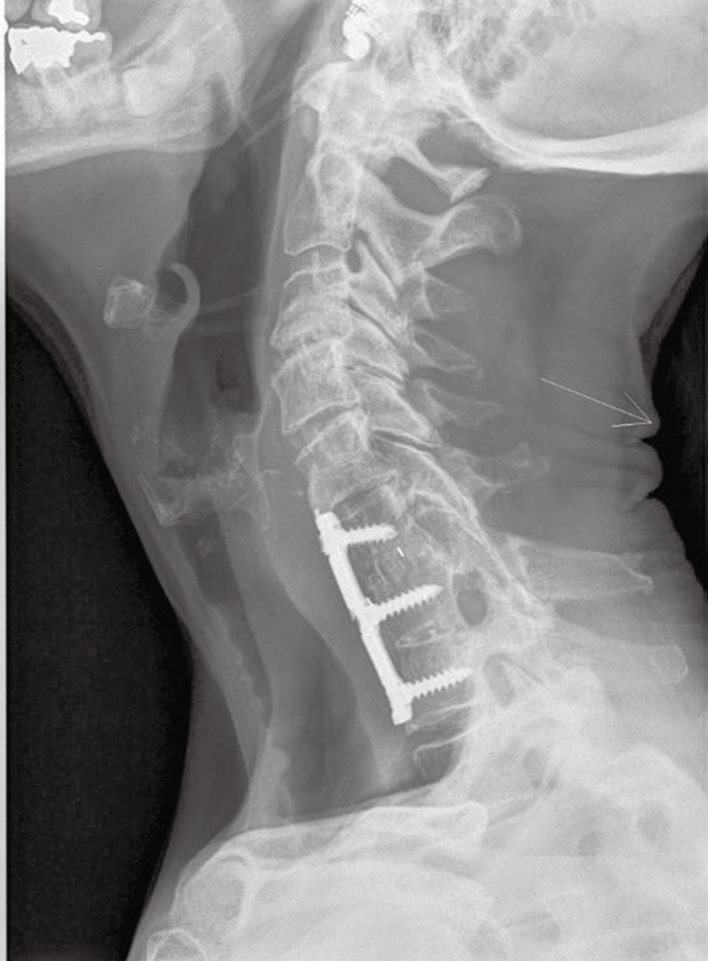
Postoperative X-rays (lateral view).

We initially performed a reduction with a Gardner-Wells tong traction. This reduction was performed under general anesthesia by pulling smoothly with the neck in mild cervical flexion under X-ray fluoroscopy, trying to pull the upper and lower articular processes “tip to tip” before to extend slowly the neck. The reduction was nearly complete but of course unstable with recurrence of the listhesis as soon as we decreased the traction force. In the same procedure, we carried out therefore an anterior discectomy and stabilization with a cage filled with bone allograft in the intersomatic space and an anterior plating as well. As the patient presented a congenital block between C7 and T1, we placed a plate extending from C6 to T1 to get a better stability and by the same time a better buttress effect (Figure 3).

She recovered fully from the tetraparesia and was able to walk freely again three days after the operation. She wore a rigid Miami cervical collar for two months and a soft collar for an additional six weeks. Plain control postoperative X-rays did not show any instability afterwards. A cervical CT scan carried out one year after the operation showed a fusion between the vertebral bodies of C6 and C7 without any sign of instability. The only complaint described by the patient, one year later, is occasional paresthesia in the central rays of the left hand.

## 3. Discussion

The literature is very rich about recent dislocations and strategies used to reduce and fuse this type of trauma. We developed 3 questions regarding the way to manage these misdiagnosed injuries.

### 3.1. The First Question Is, “Are Plain X-Rays Alone Sufficient for an Adequate Management?”

The diagnosis can be missed, even with radiological exams, if some criteria are not fulfilled. In a 32-month period study including 1331 patients having had cervical spine roentgenograms (CSRs) following blunt injury, Gerrelts et al. [1] showed that 8.2% (5 patients on 61 patients presenting a cervical spine injury) had delayed recognition of their diagnosis of fracture and/or dislocation (range from 2 to 21 days). The reason was incomplete CSRs despite multiple views (up to 13 in one case). In a retrospective study including 323 injured patients, Metak et al. [2] found 40 patients with missed injuries of the musculoskeletal system. The cervical spine was the first region affected (especially the cervicothoracic transition). The average delay of missed spine injuries was within the first 2 weeks (4 days in average). The causes were inadequate X-rays, not consistently continued diagnostic steps, inaccurate or not repeated clinical examination, and apparent insignificance of peripheral injuries in multiple-trauma patients. In another 20-year retrospective study concerning 367 patients with cervical spine injuries, Platzer et al. [3] found a diagnostic failure rate of 4.9% (18 patients). Therefore, the same author recommend further radiological (CT scan or MRI) if there is any doubt on the evaluation of the standard views.

If these two regions are not properly seen on plain X-rays or if a patient complains of neck pain with normal plain X-rays, the CT scan becomes necessary for the exclusion of cervical/cervicothoracic spine injury, according to Platzer et al. [3]. When there is a high level of uncertainty related to bony structures or ligamentous injuries in the static X-rays, dynamic flexion and extension should be avoided until the extent of the lesion is determined by CT scan or MRI [3, 4].

The usefulness of MRI to identify anterior disc herniation which is potentially dangerous during the closed reduction maneuver remains controversial. In some cases indeed, a neurological deterioration could occur after closed reduction under general anesthesia [5]. On the other hand, a closed reduction in awaken and cooperative patients may be safe without a prereduction MRI in most cases, as demonstrated by Vaccaro et al. [6]. Therefore, a prereduction MRI is recommended in unexaminable patients unable to cooperate [6, 7]. If the MRI shows a dangerous disc fragment, the wiser attitude is to carry out an open reduction (by an anterior approach) to remove the entire disc and thus the posterior disc herniation impinging the cord. This way to proceed is probably preferable despite a lack of consensus on this topic.

### 3.2. The Second Question Is, “Is Preoperative Skull Traction Useful?”

Skull traction has been proved helpful for patients with acute facet dislocations but in the literature, the success rate for initially neglected dislocations is by far much lower. In his series, Basu et al. [8] noticed that 10 of 14 patients with unilateral dislocation and only 1 of 5 patients with bilateral dislocations were successfully reduced with skull traction (mean duration of 2 days) but his patients presented a cervical dislocation since a mean duration of 14 days (7 to 21 days). Kahn et al. [9] reported a success rate of only 20% in delayed cervical facet dislocations. Hassan [10] reported a reduction of the dislocation after prolonged one-week traction in only 2 patients in his 12 patients series. Goni et al. [11] did not see any benefit from skull traction in neglected delayed distractive flexion injuries to the cervical spine after a delay of more than 3 weeks in 6 patients.

### 3.3. The Third Question Is, “What Is the Best Surgical Approach and in Case of Several Approaches, the Best Sequence?”

There is little agreement about the choice of the surgical approach and the sequence in recent dislocations: anterior alone, posterior alone, combined, and staged anterior/posterior/anterior. It depends on the presence of disc herniation, the ability of the surgeon to reduce by one approach alone. In terms of biomechanics, the combined approach is logically the more efficient in terms of stability but some studies described the anterior fixation/fusion alone as sufficient if a brace is provided to the patient [12–15].

In most cases, despite equal results in terms of fusion rate, recovery, alignment, or long-term complaints between anterior and posterior approaches [14, 16, 17], the anterior approach in acute cervical dislocations is preferred by some authors because of the risk of disc herniation impinging the cord occurring when reducing the dislocation by a posterior approach [15, 17, 18]. A vast majority of patients were reduced (in recent dislocations) by an anterior approach in a first stage [14, 17]; a second stage by a posterior approach could be added to increase the stability [17].

Little is described about delayed dislocations (more than 3 weeks old) and especially with a delay of more than one year as seen in our case. We found in the literature 9 studies concerning surgical management of patients with a neglected unreduced cervical dislocation as described thereafter.

The first study is a 12-case series (1.5 to 12 months after an accident) done by Hassan [10] who described a protocol with a one-week traction (6 to 12 kg) before the operation. The dislocation was unifacetal in two patients and bifacetal in ten. From these last ten patients with a bilateral dislocation, only 2 patients saw their dislocation reduced by the traction and were stabilized by an anterior fusion alone with plate fixation. The other 8 patients underwent a posterior partial facetectomy. If this allowed reduction (one patient), it was followed by a posterior fusion with plate fixation. If reduction was not achieved (the 7 remaining patients), the traction was carried on for another week and then followed by anterior discectomy and fusion with plate fixation. All the patients developed bone fusion and showed neurological improvement. Of course, the disadvantage of this technique is the duration of the skull traction in a supine position (1 to 2 weeks).

In a second 17-case study of delayed dislocations (range of 4 to 54 weeks; 10 unilateral dislocations and 7 bilateral dislocations), Ding et al. [19] proposed a procedure where he carried out an anterior discectomy at first to remove the fusion mass and to achieve the discectomy and filled in the space with morselized graft (from the iliac crest) in the anterior 2 thirds of the disc space without any implant. In a second posterior stage, the patient's dislocation was reduced, and the lateral masses were fixed by screws and rods. In 8 of 17 patients, there was partial bony fusion between facets which was removed to make the reduction easier. The advantages of this method are the avoidance of any risk of cord compression by a prolapsed disc using an anterior approach first and the need of only 2 stages (compared to procedures involving 3 stages). The disadvantage is an instrumentation locking only the posterior aspect of the spine but it has been proven that anterior instrumentation provides less stability than posterior fixation [12, 20].

In a third smaller series of three patients with older (>8 weeks) bilateral cervical dislocations, Bartels and Donk [21] attempted a classic anterior-posterior-anterior but failed to reduce the dislocation in the first anterior stage. So, they added an additional posterior stage resulting in an anterior-posterior-anterior-posterior sequence: anterior stage to remove the fibrocartilaginous tissue, posterior stage for screw placement, anterior stage for reduction and fusion, and eventually posterior stage for rods placement and posterior fusion. After the failures of the first two cases, they decided to carry out a posterior release first and then anterior reduction and fusion followed by posterior fusion, therefore, 3 stages in all (posterior-anterior-posterior).

In a fourth series including 13 patients, Kawano et al. [22] recommended an A-P-A method (anterior discectomy, posterior release and/or partial facetectomy, reduction, and instrumentation).

In a fifth study, Prabhat et al. [23] reviewed 15 patients and recommended a closed reduction with the patients being awake using skull traction starting at 4 kg and gradually to the maximum weight according to the vertebral level with neurological status monitoring during the course. In patients who had achieved complete closed reduction and with intact posterior ligamentous complex, only anterior cervical discectomy and fusion (ACDF) were performed, which is technically less demanding. For those patients where the closed reduction failed or having posterior ligamentous injuries, he carried out a combined procedure (anterior and posterior). He advocated a posterior-anterior-posterior (P-A-P) approach for delayed presentations of bilateral dislocations of facets except when complicated by an extruded disc where an anterior-posterior-anterior (A-P-A) is more suitable and safer.

In a sixth study, Srivastava (in a six-patient series) had the same attitude [24]: skeletal traction for 3 weeks starting with 3 kg and gradually increasing to 8-10 kg (max. 1/5^th^ of body weight). These patients where the reduction was satisfactory underwent an anterior cervical discectomy and fusion (ACDF). The other patients (where the reduction was not feasible by traction) were taken under general anesthesia, and attempt at reduction was done using skeletal traction. If the reduction was achieved, an ACDF was carried out with iliac crest autograft and anterior cervical plating. If the reduction could not be achieved by skeletal traction, the patients underwent posterior soft tissue release (with facetectomy if the open reduction was not possible). Srivastava believes that posterior release and reduction are needed when closed reduction failed and if open reduction cannot be performed, partial excision of the superior articular process of the lower vertebra has to be carried out. Once the reduction is achieved, a posterior fixation is done involving 2 levels above and 2 levels below (therefore, only a posterior surgery is achieved). But in patients having preoperative neurological deficit or younger patients (where it could be wiser to save as many motion segments as possible), a short segment fixation was done posteriorly (one level above and one level below) after being turned supine for anterior stage and ACDF (A-P).

In a seventh series including 9 patients, Liu et al. [25] recommended either the P-A method (post. release, ant. release, reduction, intervertebral grafting, and anterior plating) or the P-A-P method (the same than P-A but with post. instrumentation in a last stage).

In an eighth four-patient series, Jain et al. [26] recommended the same sequence (P-A): posterior soft-tissue release and partial facetectomy allowing partial reduction followed by interspinous wiring (Roger's technique) and autologous bone grafting of the posterior elements before the anterior stage consisting of discectomy, tricortical bone grafting, and plating.

The last and ninth study found in the literature was a case report from Payer and Tessitore with a 51 year-old patient with a C5/C6 bilateral dislocation diagnosed 10 weeks after the diagnosis [27]. The patient was operated with an anterior/posterior/anterior sequence. The first stage (anterior) was carried out to remove the fibrocartilaginous tissues between the end plates, the second stage (posterior) to reduce the dislocation and insert the lateral mass screws connected with rods, and the third and last stage (anterior) to insert the intersomatic bone-filled cage secured by an anterior plate. These two last authors believe that the two sequences (ant.-post.-ant. and post.-ant.-ant.) are adequate treatment options as in both procedures, the disc is removed before the reduction maneuver, avoiding the risk of aggravating a concomitant disc herniation through the reduction maneuver. Nevertheless, they pointed out a certain advantage in the ant.-post.-ant. sequence as the patient has to be turned only twice instead of four times in the post.-ant.-post. between the intubation and the end of the anesthesia.

## 4. Conclusion

This is a rare case of delayed presentation of an initially neglected traumatic bilateral dislocation without any neurological symptoms until a decompensation occurred with development of radicular pain in the upper limbs and progressive tetraparesia. Our attitude was to perform a reduction under general anesthesia with stabilization by an anterior approach (intersomatic cage and plate). The fact that the patient did develop neurological symptoms about one year after an asymptomatic period emphasizes the instability and indirectly the potential of reduction of the dislocation.

In these cases with neurological symptoms, we emphasize the need of a rapid surgery on a semiurgent basis (within the 24 hours) before to deal with neurological deficit and an uncertain recovery after the surgery. This is the lesson we learnt from our case with mild neurological symptoms in the beginning but deteriorating 4 days after the first presentation.

We also recommend dynamic radiographies (to exclude any unstable injuries), a CT scan if the cervicothoracic junction could not be properly seen on plan X-rays and an MRI scan to identify anterior disc herniation potentially dangerous during the closed reduction maneuver.

In this particular case, the open reduction was quite easy, and we did not find any benefit from an additional posterior procedure because of the strong buttress effect of our plate fixed in the two lower vertebral bodies (instead of one) of C7 and T1 (linked by a congenital block). This last point is definitely a matter of debate.

There is no doubt about the necessity of a surgical treatment but there is no consensus about the ideal scheme and best sequence for combined procedures in order to reduce and stabilize this delayed type of cervical trauma. Nevertheless, most authors advocated an anterior approach first in case of an extruded disc visible on the MRI to avoid any cord compression and/or neurological deficit during reduction maneuvers. It seems to be the more logical attitude in spite of a lack of consensus.

In the particular cases of unnoticed delayed dislocation, if there is no visible change in the radiological status while attempting to reduce the dislocation by external maneuvers, there is little chance to reduce it successfully only by one single approach. Therefore, for the chronic irreducible dislocations, it seems logical and safer to prepare the reduction/fusion stage (either anterior/posterior, depending on the habits and skills of the surgeon) by a first stage to carry out a release of the fibrous tissues on the opposite side (either posterior to release the facet joins or anterior to release the intervertebral disc), followed by the reduction/fusion stage itself and then by a third and last stage to lock and secure the operated level for good. Like Payer and Tessitore, we would recommend the anterior-posterior-anterior sequence (A-P-A) to turn the patient only twice instead of four times (in the P-A-P sequence) and therefore, we recommend the anterior approach first, especially in case of an extruded disc visible on the MRI.

## Data Availability

The data sets used and/or analysed during the current study are available from the corresponding author on reasonable request.
